# Ultrasound- versus landmark-guided subclavian vein catheterization: a prospective observational study from a tertiary referral hospital

**DOI:** 10.1038/s41598-019-48766-1

**Published:** 2019-08-22

**Authors:** Anna Sidoti, Etrusca Brogi, Giandomenico Biancofiore, Sergio Casagli, Fabio Guarracino, Paolo Malacarne, Lara Tollapi, Matteo Borselli, Gregorio Santori, Francesco Corradi, Francesco Forfori

**Affiliations:** 1 Department of Anaesthesia and Intensive Care, University of Pisa, Pisa, Italy; 2 Azienda Ospedaliero Universitaria Pisana, Pisa, Italy; 3 Department of Surgical Sciences and Integrated Diagnostics (DISC), University of Genoa, Genoa, Italy; 4 Department of Anesthesia and Intensive Care, Ente Ospedaliero Ospedali Galliera, Genoa, Italy

**Keywords:** Radiography, Ultrasonography

## Abstract

This was a single-center, observational, prospective study designed to compare the effectiveness of a real-time, ultrasound- with landmark-guided technique for subclavian vein cannulation. Two groups of 74 consecutive patients each underwent subclavian vein catheterization. One group included patients from intensive care unit, studied by using an ultrasound-guided technique. The other group included patients from surgery or emergency units, studied by using a landmark technique. The primary outcome for comparison between techniques was the success rate of catheterization. Secondary outcomes were the number of attempts, cannulation failure, and mechanical complications. Although there was no difference in total success rate between ultrasound-guided and landmark groups (71 vs. 68, p = 0.464), the ultrasound-guided technique was more frequently successful at first attempt (64 vs. 30, p < 0.001) and required less attempts (1 to 2 vs. 1 to 6, p < 0.001) than landmark technique. Moreover, the ultrasound-guided technique was associated with less complications (2 vs. 13, p < 0.001), interruptions of mechanical ventilation (1 vs. 57, p < 0.001), and post-procedure chest X-ray (43 vs. 62, p = 0.001). In comparison with landmark-guided technique, the use of an ultrasound-guided technique for subclavian catheterization offers advantages in terms of reduced number of attempts and complications.

## Introduction

The subclavian vein (SCV) is a common site of percutaneous access for central vein cannulation in intensive care. This site offers several advantages, including a lower incidence of thrombosis and central venous catheter (CVC)-related sepsis, with better patient comfort and easier nursing care^1–3^. SCV is an alternative to internal jugular vein (IJV) when this is difficult to locate, as in hypovolemic or obese patients^4,5^. SCV cannulation has the advantage of fixed landmarks but may be associated with potentially severe complications, e.g., pneumothorax or hemothorax, likely related to limited operator experience^6^.

The role of ultrasound (US) for IJV catheterization has been accepted as the standard of care after the recommendations by National Institute for Health and Clinical Excellence (NICE) in 2002, but the same guidelines stated that there was insufficient evidence to support the use of US for SCV catheterization. A Cochrane systematic review published in 2015 concluded that “two-dimensional US offers small advantages in terms of safety and quality in comparison with an anatomical landmark technique for either subclavian or femoral vein cannulation”^8^. On the other hand, in recent years, several trials have shown a reduction in complications and an improvement in first-pass success when US were used^9–12^.

In our center, after long-standing experience with US-guided IJV and femoral vein cannulation, we have extended this technique to SCV cannulation with encouraging results. The goals of present observational study were to compare, in two groups of adult patients, the effectiveness and safety of SCV cannulation with US- or landmark-guided technique.

## Methods

After approval by the Local Research Ethics Committee (number 23185, May 2018), a single-center, observational, prospective study took place at Pisa University Hospital from May 1 to July 30, 2018. Clinical Trials registration number was NCT03207932. Informed consent was obtained from all conscious patients. Unconscious patients were included after assessment of the benefit-to-risk ratio with the attending physician and consent was obtained when the patient regained consciousness. If a patient had not regained consciousness or died, consent was waived in accordance with the directives of our ethics committee. The study was done in accordance with the Declaration of Helsinki^13^ and the applicable STROBE guidelines.

### Study population and protocol

We recruited 148 critically ill adults requiring CVC positioning (Table 1). One group included 74 consecutive patients admitted to intensive care unit and studied by using a US-guided technique, another group included 74 consecutive patients admitted to emergency department and studied by using a landmark-guided technique. Indications for CVC insertion were the following: hemodynamic monitoring, treatments with vasopressors or any drug likely to induce phlebitis, temporary cardiac pacemaker and hemodialysis. Life-threatening conditions requiring an emergency CVC positioning were exclusion criteria.

**Table 1:** Comparison between ultrasound-guided and landmark groups for demographics and admission data.

Variable	US group (n = 74)	LM group (n = 74)	P value
Age (years)	64 ± 13	60 ± 19	0.07
Sex			
Male	28 (37%)	54 (72%)	<0.001
Female	46 (62%)	20 (27%)	<0.001
Weight (Kg)	73.6 ± 22.5	78.3 ± 14.7	0.160
BMI (Kg/m^2^)	26 ± 6.6	25.8 ± 6.5	0.380
Admission diagnosis			
Medical	49 (66%)	26 (35%)	<0.001
Surgical	23 (31%)	16 (21%)	0.510
Trauma	2 (3%)	31 (41%)	<0.001

US: ultrasound-guided. LM: landmark, BMI: body mass index. Data are presented as mean ± standard deviation or as count and percentage (%) where appropriate.

All catheterizations were done by anesthesiologists with 3-to-6 years’ experience of more than 50 SCV cannulations per year, approximately half landmark and half US guided

### Data collection

The following variables were recorded: site of cannulation (right or left SCV), first placement success rate, number of attempts, mechanical ventilation withdrawal, cannulation failure, reason for site change, pre- and post-procedural check (ultrasound, bubble test, chest X-ray) and type of complications.

### CVC technique

**Figure 1:**
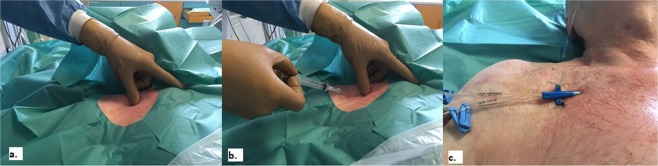
Landmark infra-clavicular approach to cannulate subclavian vein. Infra-clavicular approach was used in landmark approach, with landmark points being the clavicle (the “break” or transition point, which is the junction of the medial one-third and lateral two-thirds of the clavicle) and the sternal notch. (**a**) The needle should pass below the clavicle and above the first rib, with appropriate point for cutaneous puncture being 1–2 cm below and laterally to the clavicular transition point. (**b**) The needle is advanced parallel to the floor, through the subclavian muscle, until it enter the subclavian vein.

The lateral subclavian vein, which is the extension of the axillary vein beyond the junction with the cephalic vein, was highlighted by the acoustic shadow of the first rib^14–16^. In the landmark group, an infra-clavicular approach was used^17^, with landmark points being the clavicle (the “break” or transition point, which is the junction between the medial one-third and lateral two-thirds of the clavicle) and the sternal notch. The needle was passed below the clavicle and above the first rib, with the appropriate point for cutaneous puncture being 1–2 cm below and laterally to the clavicular transition point. The needle was advanced parallel to the floor, through the subclavian muscle, until it entered the subclavian vein (Fig. 1).

**Figure 2:**
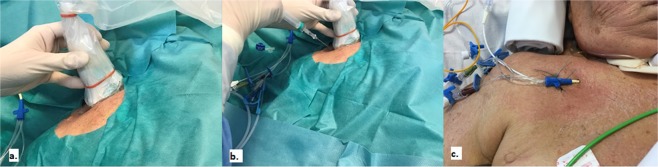
Ultrasound- guided subclavian cannulation using in-plane approach. Subclavian and axillary veins were visualized by placing a linear transducer in the infra-clavicular fossa, to obtain a short axis view of vein and artery; then, the transducer was rotated until a longitudinal view was obtained (**a**) and tilted in order to visualize the vessel until its disappearance below the clavicle. In the longitudinal orientation, the needle was inserted at transducer midpoint providing an in-plane orientation (**b**).

**Figure 3:**
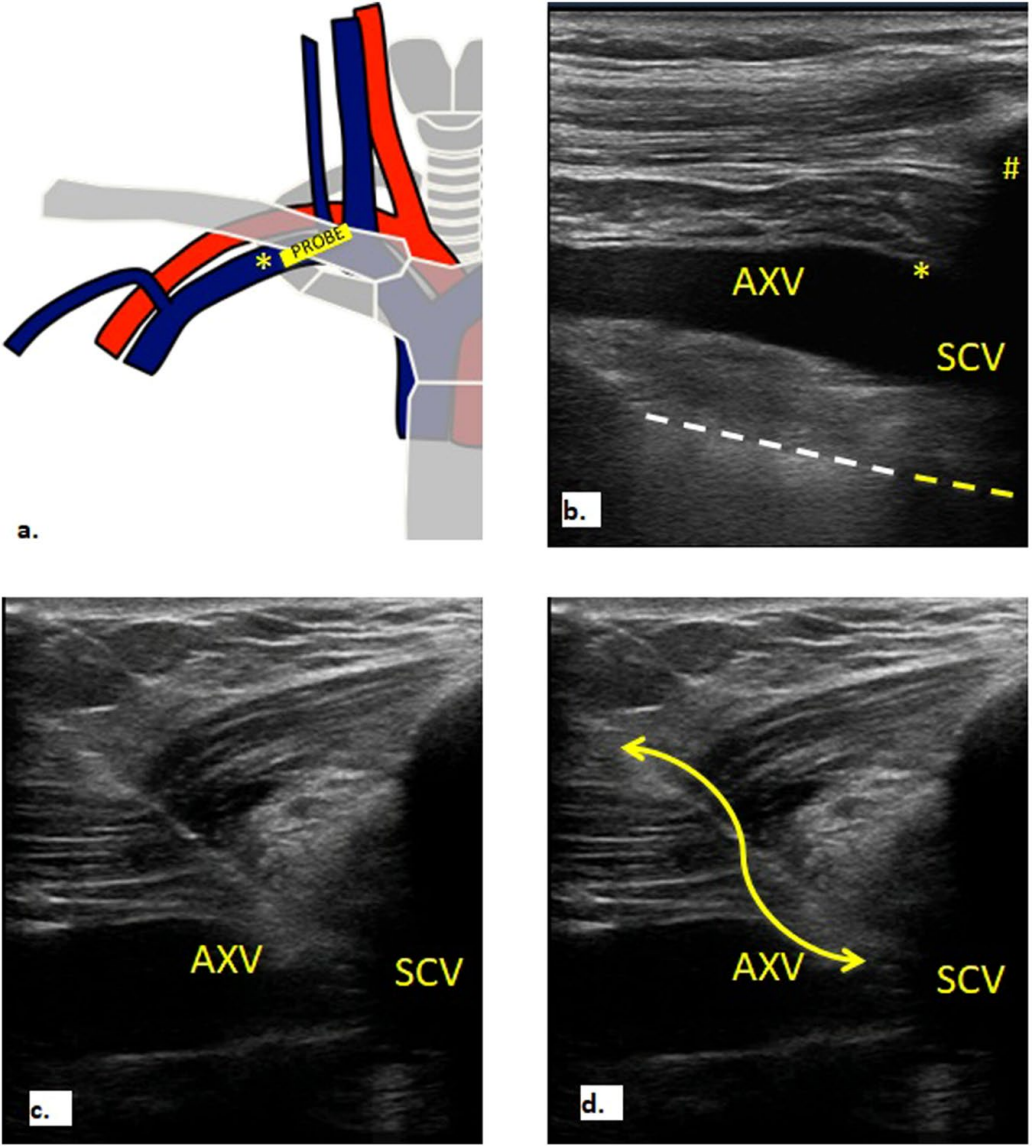
Probe positioning and ultrasound visualization of axillary vein, subclavian vein and pleural line before puncture (**a,b**). Real-time visualization of needle inserted in subclavian vein (**c,d**). In (**a**) linear probe is positioned in infra-clavicular fossa in-line with subclavian vein to obtain longitudinal view. *Yellow asterisk* marks transition from axillary to subclavian vein in (**a**) and (**b**). In (**c,d**) the needle was advanced slowly and its tip visualized throughout the procedure while maintaining a view of the vessel that was finally entered at its lateral border, just before the acoustic shadow of the clavicle, marks with *yellow hash* in (**b**), and far from the cephalic vein confluence. Pleural line is visualized throughout the procedure, it is marked with *white dotted* line in (**b**). *Yellow dotted line* marks the acoustic shadow of first rib (**b**).

In the US-guided groups, we used a longitudinal “in-plane” approach^6,18^. Subclavian and axillary veins were visualized by placing a linear transducer in the infra-clavicular fossa, to obtain a short axis view of vein and artery; the transducer was then rotated until a longitudinal view was obtained and tilted until it disappeared below the clavicle in order to visualize the vessel (Fig. 2). This view enabled the visualization of the transition from medial axillary to lateral subclavian vein and the pleural line, thus enabling to visualize lung sliding and identify pre- and post-procedural pneumothorax, if any. In the longitudinal orientation, the needle was inserted at transducer midpoint providing an in-plane orientation. The needle was advanced slowly and its tip visualized throughout the procedure while maintaining a view of the vessel that was finally entered at its lateral border, just before the acoustic shadow of the clavicle and far from the cephalic vein confluence. After the needle-puncture of the vessel, the guidewire was inserted and visualized in real time (Fig. 3).

Cannulation failure was defined as a change in cannulation site due to ensuing complication or unsuccessful attempts. Subsequent attempts were made at site different from subclavian vein (jugular or femoral) by using the same cannulation technique, i.e., landmark- or US-guided.

Correct catheters placements were checked by post-procedural chest X-ray or US bubble test, the choice between these being based on clinical judgment.

US bubble test was performed after CVC insertion as originally described^19^ and currently recommended^20^. Both, B-mode ultrasound and contrast enhanced US (CEUS) were performed. First, conventional B-mode ultrasound was used to examine both SV and IJV. Then, the heart was visualized through the epigastric and subcostal acoustic windows along the short heart axis, in order to see both cava veins and the right atrium at the same time confirming catheter placement. Catheter misplacement was defined as the CVC tip in the right atrium or in a vein other than superior vena cava (SVC) or SVC-to-right atrium junction. The catheter tip identification in the right atrium was confirmed by CEUS, using an air-blood-saline mixture containing 8 mL of saline, 1 mL of blood and 1 mL of air. The homogeneous solution thus obtained, was rapidly injected as a bolus. The test was deemed positive for correct CVC placement when real-time CEUS recorded a laminar jet flow of multiple microbubbles flowing from the SVC within 1 to 2 seconds after the start of injection. The test was deemed negative when the catheter tip was seen in the atrium or inferior vena cava or when the real-time CEUS recorded a turbulent flow coming from the atrium or inferior vena cava.

### Statistical analysis

To calculate the sample size for SCV cannulation, we followed the recommendations of Kim and Seo^21^, assuming a statistical power of 0.90 (alpha = 0.05) with success rates of 100% using US 87.5% using landmark technique^22^. The precise sample size estimation was 74 per group and 148 in total.

Data were expressed as mean ± SD or as count and percentage (%) when appropriate. Success rate and 95% confidence interval (CI) were calculated for the cumulative cannulation attempts. Unpaired Student’s t test, chi-square or Fisher’s exact test were used to identify significance of differences between groups. A two-sided p value < 0.05 was considered significant. SPSS software (version 11.0; SPSS Inc, Chicago, IL) was used for all statistical analyses.

### Ethics approval and consent to participate

Local Research Ethics Committee of Pisa University Hospital approved the present study (approval number 23185, May 2018). Written informed consent was obtained from all the participants in the study. Procedure was carried out in accordance with the Declaration of Helsinki^13^.

## Results

Anthropometric characteristics were not different between groups (Table 1). Admissions for medical reasons were more frequent in US than landmark-guided group (66 vs. 35%, p < 0.001) and for trauma more frequent in the latter (31 vs. 3%, p < 0.001).

In both groups, there was a preference for right-site cannulation, which was chosen in 69% and 77% of US-guided and landmark-guided procedures, respectively.

Despite similar success rates between groups, i.e., 96% in US-guided and 92% in landmark-guided group (Table 2), the success at first attempt was more frequent in the former (86.5 vs 40%, p < 0.001). Furthermore, we found that in ultrasound-guided group the mean number of attempts was significantly (p < 0.001) lower than in landmark group, i.e., 1.14 (0.40) vs 2.08 (1.29).

**Table 2:** Comparison between ultrasound-guided and landmark groups for success rate, cannulation attempts, and complication’s type.

Results of Cannulation	US group (n = 74)	LM group (n = 74)	P value
Success rate			
Feasibility	71/74 (96%)	68/74 (92%)	0.494
First pass success rate without complication	64(86.5%)	30 (40%)	<0.001
Cannulation site			
Left Subclavian	23 (31%)	17(23%)	0.355
Right Subclavian	51 (69%)	57 (77%)	
Cannulation Attempts	1.14 ± 0.4	2 ± 1.29	<0.001
Failure Rate	3 (4%)	6 (8.1%)	0.112
Mechanical complication rate (Global)	2 (2.7%)	13 (17.5%)	<0.001
Mechanical complication type			
Pneumothorax	1 (1.35%)	2 (2.7%)	0.591
Arterial puncture	0	5 (6.7%)	0.018
Hematoma	0	2 (2.7%)	0.205
Hemothorax	0	0	—
Difficult to pass	0	0	—
Seldinger	0	2 (2.7%)	0.205
Malposition	0	2 (2.7%)	0.205

US: ultrasound-guided. LM: landmark. Data are presented as mean ± standard deviation or as count and percentage (%) where appropriate.

Mechanical complications were significantly less in US- than landmark-guided group (3/74 vs 13/74, p < 0.001). Arterial punctures of subclavian artery occurred in five patients of landmark-guided but none of US-guided group (p = 0.018), whereas pneumothorax occurred with similar frequency in both groups (p = 0.591).

Concerning post-procedure controls (Table 3), more chest X-rays were necessary in landmark- than US-guided group (83 vs 58% p < 0.001). US scanning was necessary in 20% of landmark group, 10% before and 20% after cannulation. As expected, in US-guided group there were more US scans than in landmark-guided group, either before (94% vs 10%, p < 0.001) or after cannulation (55% vs 20%, p < 0.001). Correct cannulation was proved with bubble test in more patients of US- than landmark-guided group (44 vs 17%, p < 0.001). Mechanical ventilation was interrupted in 77% of patients in landmark- vs 1.3% of those of US-guided group (p < 0.001).

**Table 3:** Other parameters evaluated during catheterisations in the ultrasound-guided and landmark groups.

Variable	US group (n = 74)	LM group (n = 74)	P value
ECBC	70 (94%)	8 (10%)	<0.001
ECAC	41 (55%)	15 (20%)	<0.001
Bubble Test	33 (44%)	12 (17%)	<0.001
Chest X-ray	43 (58%)	62 (83%)	0.001
MV	29 (39%)	65 (87%)	<0.001
Interruption of MV during catherization	1 (1.3%)	57 (77%)	<0.001

US: ultrasound-guided. LM: landmark. ECBC: echography control before cannulation. ECAC: echography control after cannulation. MV: mechanical ventilation. Data are presented as mean ± standard deviation or as count and percentage (%) where appropriate.

## Discussion

Our analysis demonstrated that US guidance significantly reduced the adverse events of SCV cannulation, though the overall success rate was not significantly different from landmark technique. This result is in line with a recent systematic review showing clear advantages for US-guided versus anatomic landmark-guided techniques at cannulation site, including fewer complications and higher success rates^11^. Real-time US guidance for vein cannulation is supported by increasing evidence^7^ and has been recommended to be used routinely for CVC insertion at any site^20^. Nevertheless, its systematical use still faces barriers and resistance especially in particular clinical settings, like emergency departments where the US availability is still a limiting factor^23^.

As expected, the need of chest X-ray to confirm correct catheter placement was less in US-guided group, thus with less radiation exposure and costs, though not quantified in the present study. In our previous study, we demonstrated a reduction of chest X-ray after introduction of pulmonary US in intensive care routine, a change that reduced costs by 57% without affecting outcomes^24^.

The catheter placement was confirmed by US-based bubble test more frequently in the US-guided group, thus avoiding the use of chest X-ray. On the contrary, chest X-ray was the preferred confirmatory method in the landmark-guided group.

Another advantage of using US is the possibility to verify vein patency, anatomic variations, artery and pleura locations before cannulation in a systematical approach, which allows making correct choice of cannulation site and reducing of adverse events^25^.

Invasive mechanical ventilation in patients during the procedures was stopped in < 2% of cases in the US-guided but in 77% of those in the landmark-guided group, because the visualization of pleura made it unnecessary. There was no evidence of efficacy in the interruption of mechanical ventilation in terms of lower incidence of pneumothorax, but it has been shown that it may cause hypoxemia in critically ill patients^25^.

This study has several limitations. First, the study was not randomized, so it was impossible to standardize the procedures. Second, patients’ characteristics were different, with the landmark-guided procedure used more frequently in emergency department where clinical conditions, technical and human resources are more demanding and with a significant higher number of trauma patients. Third, we did not obtain the measurement of time to complete the procedures.

## Conclusions

In this observational study, representative of the every-day clinical practice in a large tertiary Italian hospital, the subclavian vein cannulation with US-guided technique has been confirmed to provide better results than landmark-guided technique in critical care patients, with a lower incidence of complications and higher first-pass success rate. In the light of the growing evidence of efficacy and safety of the subclavian vein cannulation with US guidance, we hope that its use may be extended in the future even to emergency settings.

## Data Availability

Please contact author for data requests
